# Spatial Epidemiological Analysis of Keshan Disease in China

**DOI:** 10.5334/aogh.3836

**Published:** 2022-09-12

**Authors:** Yuehui Jia, Shan Han, Jie Hou, Ruixiang Wang, Guijin Li, Shengqi Su, Lei Qi, Yuanyuan Wang, Linlin Du, Huixin Sun, Shuxiu Hao, Chen Feng, Yanan Wang, Xu Liu, Yuanjie Zou, Yiyi Zhang, Dandan Li, Tong Wang

**Affiliations:** 1 Institute of Keshan Disease, Chinese Center for Endemic Disease Control, Harbin Medical University, Harbin 150081, China; 2 Infectious Disease Hospital of Heilongjiang Province, Harbin 150081, Heilongjiang, China; 3 School of Public Health and Management, Binzhou Medical University, Yantai 264003, China; 4 Yidu Central Hospital of Weifang, Weifang 262500, China; 5 Ningbo Municipal Center for Disease Control and Prevention, Ningbo 315010, China; 6 Yantai Disease Prevention and Control Center, Yantai 264003, China

**Keywords:** Keshan disease, spatial epidemiology, precision prevention and control, spatial autocorrelation, spatial regression

## Abstract

**Objectives:**

Few researchers have studied the national prevalence of Keshan disease (KD) in China using spatial epidemiological methods. This study aimed to provide geographically precise and visualized evidence for the strategies for KD prevention and control.

**Methods:**

We surveyed and analyzed 237,000 people in 280 out of 328 KD-endemic counties (85.4%) in mainland China using a design of key investigation based on case-searching in 2015–2016. ArcGIS version 9.0 was used for spatial autocorrelation analysis, spatial interpolation analysis and spatial regression analysis.

**Results:**

Global autocorrelation analysis showed that global clustering of latent Keshan disease (LKD) prevalence was noted (Moran’s *I* = 0.22, *Z* = 7.06, and *P* < 0.0001), no global clustering of chronic Keshan disease (CKD) prevalence (Moran’s *I* = 0.03, *Z* = 1.10, and *P* = 0.27) was observed. Spatial regression analysis showed that LKD prevalence was negatively correlated with per capita disposable income (*t* = –4.36, *P* < 0.0001). Local autocorrelation analysis at the county level effectively identified the cluster areas of LKD prevalence in the provinces of Shaanxi, Gansu, Shanxi, Inner Mongolia, and Jilin. The high-high cluster areas should be given priority for precision prevention and control of Keshan disease.

**Conclusions:**

This spatial epidemiological study revealed that LKD prevention and control should be strengthened in areas with high values of clustering. Our findings provided spatially, geographically precise and visualized evidence for prioritizing KD prevention and control.

## 1. Introduction

Keshan disease (KD) is an endemic cardiomyopathy that mainly occurs in low-selenium areas in mainland China [1,2]. Keshan disease has the characteristics of obvious endemic, seasonal and population-based occurrence in epidemiology. Keshan disease has been prevalent in 328 counties in 16 provinces in mainland China, mostly occurs in severe cold winters in northern endemic areas, mostly occurs in hot summers in southwestern endemic areas, and mainly affects children aged at 2 to 10 and women of child-bearing age [3,4,5]. The etiology of Keshan disease seems quite complicated. It is well recognized that Keshan disease is strongly associated with selenium deficiency. The geographical distribution of Keshan disease-endemic regions was highly overlapped with the low-selenium geological belt in the observational epidemiological studies, and selenium supplementation can effectively decrease the incidence of Keshan disease in the interventional studies. In addition, several studies reported that Keshan disease has a relationship with dietary nutritional factors (vitamin E, protein, or amino acid deficiency) and infection (virus, particularly Coxsackie B viruses of enteroviruses, and mycotoxins), though there has been little evidence of interventional studies [6,7,8,9]. However, it should be emphasized that the strong association of Keshan disease with selenium deficiency does not mean that selenium deficiency is the full and only cause(s) of Keshan disease. Although presently Keshan disease has been well controlled in most endemic areas and its incidence has significantly declined, chronic Keshan disease (CKD) and latent Keshan disease (LKD) exist and still endanger the health of people living in endemic areas [10,11]. Clinically, the onset of CKD is slow. CKD patients are characterized by chronic heart failure, dilated cardiac chambers, cardiomegaly, and thinning of the heart walls [12]. The cases of LKD are found only in surveys. The onset of LKD is disguised, with few signs and symptoms, and the cardiac function of the patients is reasonably good in the compensatory stage. The typical presentations of the electrocardiogram of LKD patients are ventricular extrasystole and right bundle branch block or ST–T changes. Cardiomegaly is not observed [12]. Therefore, Keshan disease remains a public health problem that cannot be ignored [13].

Few researchers have studied the prevalence of Keshan disease using spatial epidemiological methods. Thus, a comprehensive spatial description and analysis of Keshan disease in China is lacking [14,15,16]. Furthermore, the early stage of Keshan disease surveillance is limited in terms of funding and sample size, limiting statistical referral. Since 2013, the work for Keshan disease prevention and control has been in the stage of elimination and assessment, and most KD-endemic counties in China have been included in the national KD surveillance, establishing a foundation for carrying out a spatial epidemiological study [17,18,19,20,21]. Spatial epidemiology is mainly used to comprehensively describe and analyze diseases according to their geographic information, and its results could be used as spatially precise and visualized evidence for prioritizing the keys for prevention and control as well as assessing the effectiveness of those measures [22,23,24,25,26,27,28,29].

Spatial analysis is thus very suitable for the study of an endemic disease such as Keshan disease. Prevalence is the most important indicator for assessing the epidemic status and the effectiveness of prevention, control, and elimination of Keshan disease. It is essential to carry out spatial epidemiological analysis of KD prevalence to explore whether Keshan disease is spatially clustered and to analyze the clustering characteristics in order to provide geographically visualized evidence of spatial epidemiology for KD prevention and control.

## 2. Methods

### 2.1. Study design

This study was conducted in KD-endemic counties in mainland China using the method of key investigation based on case-searching [30]. Two endemic townships with the most cases were selected from each endemic county, and one survey site (village) in each endemic township was investigated.

### 2.2. Participants

**Figure 1:**
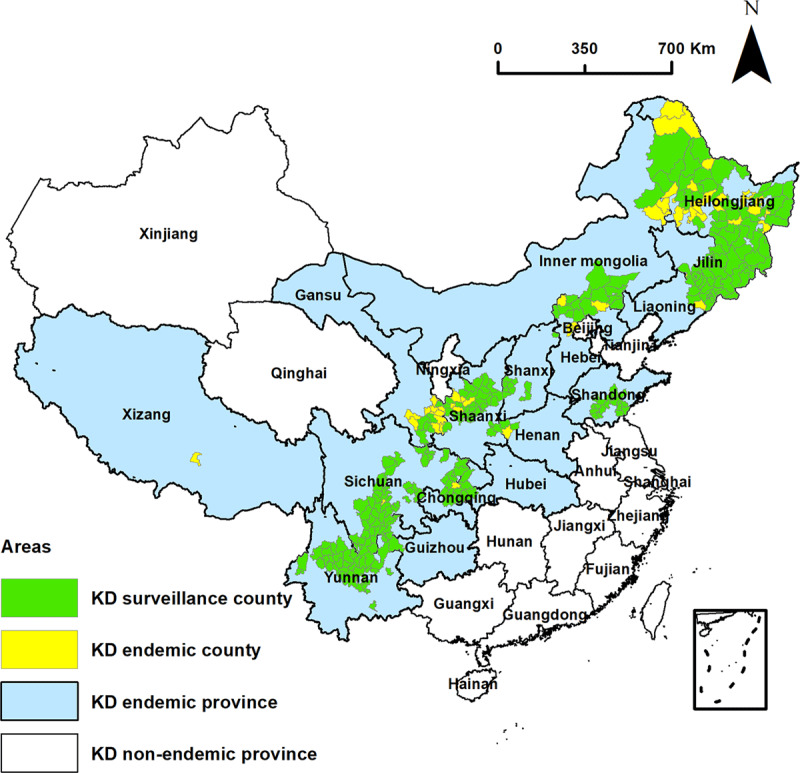
The spatial distribution of KD endemic areas.

During 2015–2016, we surveyed 237,000 individuals from 280 out of 328 KD-endemic counties (85.4%) in 15 KD-endemic provinces in mainland China and conducted a spatial analysis of KD prevalence at the county level. The inclusion criteria were that the participants had lived in their residence for not less than 6 months in the past 12 months. The spatial distribution of KD endemic areas is shown in Figure 1.

### 2.3. Diagnostic criteria

Keshan disease diagnosis was confirmed according to the Criteria for Diagnosis of Keshan Disease (WS/T 210-2011) [1].

Chronic KD: the onset is slow. The typical clinical presentations of the patients are chronic heart failure, dilated cardiac chambers, cardiomegaly, and the heart walls become thinner than normal; and there is widespread myocardial fibrosis. According to the New York Heart Association (NYHA) Functional Classification classes II, III, and IV, the CKD is further classified into Chronic II, Chronic III, and Chronic IV, respectively.

Latent KD: the onset is disguised. The cardiac function of the patients is reasonably good in the compensatory stage (NYHA class I). The typical presentations of the electrocardiogram are ventricular extrasystole and right bundle branch block or ST–T change. Cardiomegaly is not observed.

### 2.4. Definition of a KD-endemic area

KD-endemic areas were confirmed according to the Keshan Disease Endemic Area Definition and Classification (GB 17020-2010) [31].

### 2.5. Economic data

Economic data of per capita disposable income, obtained from the China Statistical Yearbooks 2016–2017, were mainly surveyed in KD-endemic areas.

### 2.6. Statistical analysis

Epi Info version 3.5.1 was used for data entry and management, SPSS version 17.0 for data cleaning, and ArcGIS version 9.0 for spatial analysis, including spatial autocorrelation analysis, spatial interpolation analysis, and spatial regression analysis. Global Moran’s index (Moran’s *I*) was used for global autocorrelation analysis, and the spatial distribution characteristics of KD prevalence were investigated from the overall level to determine whether spatial clustering existed among surveillance sites in each endemic county. Local Moran’s *I* and the Getis-Ord Gi^*^ statistic were used for local autocorrelation analysis to explore the specific cluster areas for LKD prevalence in China. The corresponding *Z* values of 90%, 95%, and 99% confidential interval (CI) for the Getis-Ord Gi^*^ were ±1.65, ±1.96, and ±2.58, respectively. Inverse distance weighted method was used for spatial interpolation analysis to estimate LKD prevalence in KD endemic areas not included in the national KD surveillance, and a predictable map of the spatial distribution of LKD prevalence in all endemic counties in China was created. Finally, the ordinary least squares (OLS) method was used for spatial regression to analyze the factors of LKD or CKD prevalence. The test level of alpha was set at 0.05 (two-sided), and *P* < 0.05 was considered statistically significant.

## 3. Results

### 3.1. Spatial Distribution of the Study Participants

**Figure 2:**
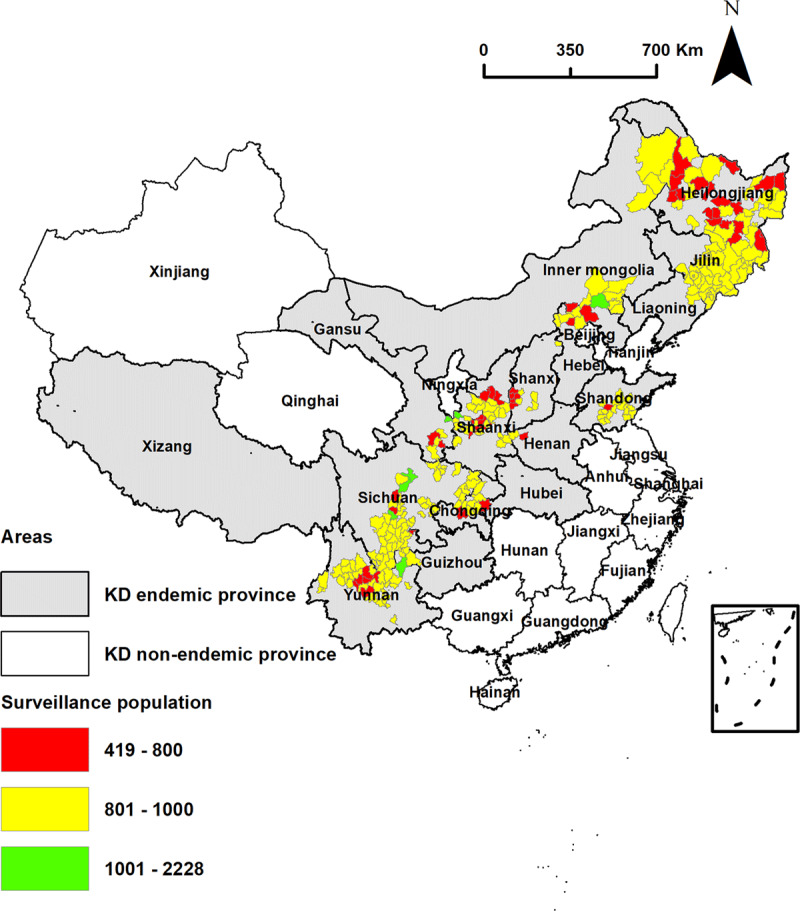
The spatial distribution of the study population in KD endemic areas.

A total of 237,000 individuals from 280 out of 328 KD-endemic counties (85.4%) in 15 KD-endemic provinces in mainland China were surveyed. The spatial distribution of the study population in KD endemic areas by county is shown in Figure 2.

### 3.2. Global autocorrelation analysis

**Figure 3:**
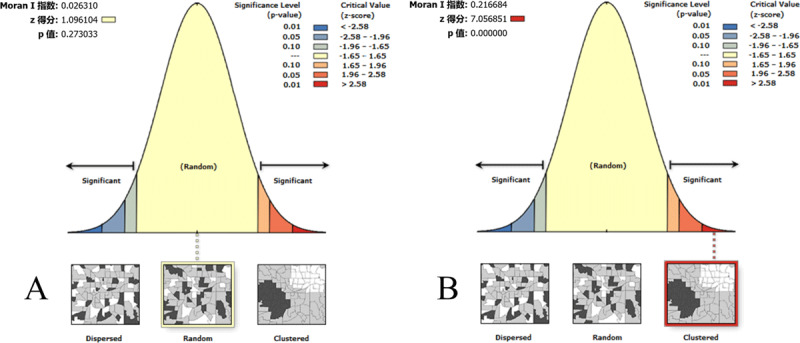
**Global spatial autocorrelation analysis of CKD and LKD prevalence in China. A)** CKD prevalence; **B)** LKD prevalence. The left side of the figure represents dispersed areas, the right side represents clustered areas, and the middle represents random areas.

The results of the global autocorrelation analysis of CKD prevalence were not significant (Moran’s *I* = 0.03, Z = 1.10, and *P* = 0.27), suggesting that CKD prevalence was likely randomly distributed and was not globally clustered, as shown in Figure 3A. Meanwhile, the results for LKD prevalence were significant (Moran’s *I* = 0.22, Z = 7.06, and *P* < 0.0001), indicating that LKD prevalence had a positive spatial autocorrelation and was globally clustered, as shown in Figure 3B.

### 3.3. Local autocorrelation analysis

#### 3.3.1. Local Moran’s I analysis

**Figure 4:**
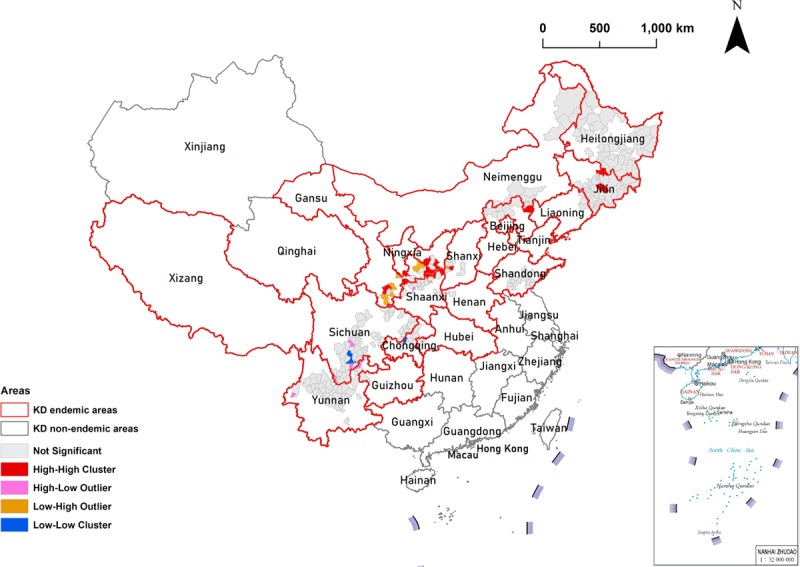
**Clusters identified by local Moran’s *I* analysis for LKD prevalence by county in China.** Red borders in the spatial thematic map represent KD-endemic areas.

High-high (H-H) cluster areas were detected in most counties of Shaanxi Province in northwestern China and in a few counties of Shanxi Province and the Inner Mongolia Autonomous Region in northern China, Jilin Province in northeastern China, and Gansu Province in northwestern China (total of 13 cluster counties). H-H cluster areas indicated that the high values of LKD prevalence were clustered among neighboring counties (positive correlation). High-low (H-L) cluster areas were detected in the counties of Sichuan and Yunnan Provinces in southwestern China and Shaanxi Province in northwestern China (a total of 6 cluster counties). H-L cluster areas indicated that counties with high values of LKD prevalence were surrounded by those with low prevalence (negative correlation). Low-high (L-H) cluster areas were detected in most counties of Gansu Province in northwestern China and in a few counties of Shanxi Province in northern China and Shaanxi Province in northwestern China (a total of 8 cluster counties). L-H cluster areas indicated that counties with low values of LKD prevalence were surrounded by those with high prevalence (negative correlation). Lastly, low-low (L-L) cluster areas were detected in the counties of Sichuan and Chongqing Provinces in southwestern China (a total of 3 cluster counties). These L-L cluster areas indicated that the low values of LKD prevalence were clustered among neighboring counties (positive correlation). The details are shown in Table 1 and Figure 4.

**Table 1:** Clusters identified by local Moran’s *I* analysis for LKD prevalence by county in China.


TYPE OF CLUSTERING	PROVINCE	COUNTY

H-H clustering	Shaanxi	Long, Baota, Zhidan, Fu, Luochuan, Yichuan, Huangling

	Shanxi	Pu

	Inner Mongolia	Ningcheng

	Jilin	Huadian, Shulan

	Gansu	Kongtong, Li

H-L clustering	Sichuan	Renhe, Hanyuan, Butuo

	Yunnan	Yongshan, Lianghe

	Shaanxi	Qishan

L-H clustering	Gansu	Qinzhou, Zhuanglang, Qingcheng, Huachi, Wudu, Cheng

	Shanxi	Daning

	Shaanxi	Wangyi

L-L clustering	Sichuan	Zhaojue, Yuexi

	Chongqing	Dianjiang


#### 3.3.2. Local Getis-Ord Gi* analysis

**Figure 5:**
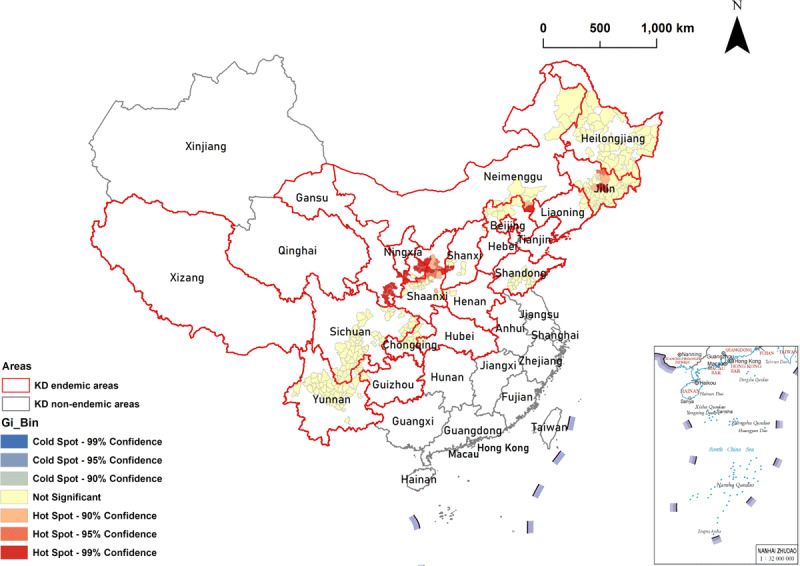
Clusters identified by Local Getis-Ord Gi^*^ analysis for LKD prevalence by county in China. Red borders in the spatial thematic map represent KD-endemic areas. Colors in the spatial thematic map represent hot spots and cold spots of spatial clustering with 90% CI, 95% CI, and 99% CI.

The hot spots of LKD prevalence were mainly concentrated in most counties of Shaanxi and Gansu Provinces in northwestern China and in a few counties in Shanxi Province and the Inner Mongolia Autonomous Region in northern China and Jilin Province in the northeast central region of China. A total of 27 hot spots were detected, which indicated that the high values of LKD prevalence were clustered in these areas. No cold spots were observed. Details are shown in Table 2 and Figure 5.

**Table 2:** Clusters identified by Local Getis-Ord Gi^*^ analysis for LKD prevalence by county in China.


TYPE OF CLUSTERING	PROVINCE	COUNTY

Hot spot 99% CI	Shaanxi	Long, Zhidan, Fu, Yichuan

	Gansu	Qinzhou, Kongtong, Zhuanglang, Huating, Qingcheng, Huachi, Heshui, Wudu, Cheng, Xihe, Li

	Shanxi	Ji, Daning, Pu

	Inner Mongolia	Ningcheng,

	Jilin	Huadian

Hot spot 95% CI	Shaanxi	Changwu, Bin, Baota, Luochuan, Huangling

	Gansu	Zhengning

	Jilin	Shulan

Hot spot 90% CI	Shaanxi	Wangyi, Xunyi, Hua, Ansai, Ganquan, Huanglong

	Jilin	Jiaohe, Panshi

	Inner Mongolia	Kelaqinqi


### 3.4. Spatial interpolation analysis

**Figure 6:**
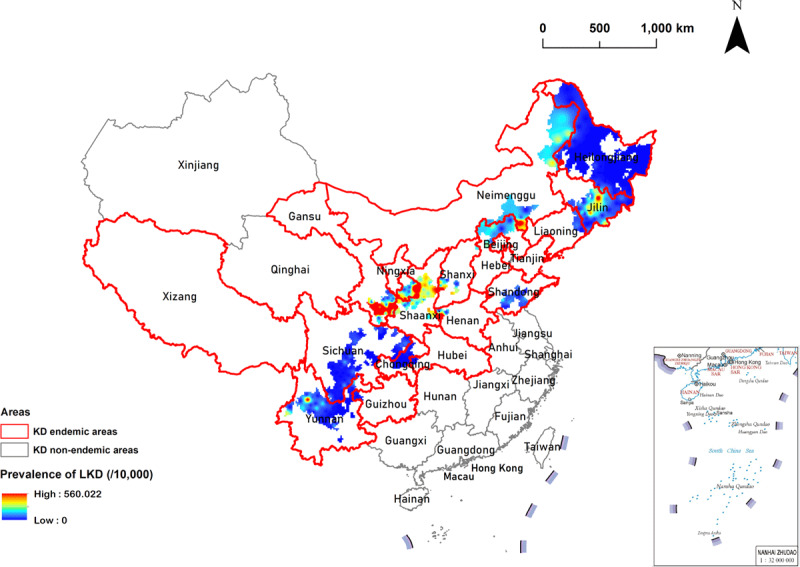
**Spatial interpolation analysis of LKD prevalence in China.** Red borders in the spatial thematic map represent KD-endemic areas. The blue to red colors in the spatial thematic map indicate gradual increases in LKD prevalence.

The results in Figure 6 show a predictable map of the spatial distribution of LKD prevalence in all 328 endemic counties in China.

### 3.5. Spatial regression analysis

A spatial regression model was developed with LKD or CKD prevalence as the dependent variable and per capita disposable income as the independent variables. The results of the LKD model were significant (*F* = 10.00, *P* < 0.001), and the regression coefficient *R*-squared (*R*^2^) and adjusted *R*-squared (*R*^2^) of the model were 0.1205 and 0.1085, respectively. The residual value of the LKD model was independent, and no spatial autocorrelation was observed (Moran’s *I* = 0.07, *P* = 0.76). The LKD prevalence was significantly negatively correlated with per capita disposable income, and the prevalence of LKD decreased by 0.0099 for each unit increase in per capita disposable income. The results of the CKD model were not significant (*F* = 1.89, *P* = 0.1553), and the regression coefficient *R*-squared (*R*^2^) and adjusted *R*-squared (*R*^2^) of the model were 0.0252 and 0.0118, respectively. The residual value of the LKD model was independent, and no spatial autocorrelation was observed (Moran’s *I* = 0.04, *P* = 0.65). There was no significant correlation between CKD prevalence and per capita disposable income (*t* = –1.58, *P* = 0.1170), and the prevalence of CKD decreased by 0.0006 for each unit increase in per capita disposable income. The details are shown in Table 3.

**Table 3:** Spatial regression analysis of LKD and CKD prevalence.


CHARACTERISTIC	REGRESSION COEFFICIENTS	STANDARD DEVIATION	*t*	*P* VALUE

LKD				

Per capita disposable income	–0.0099	0.0023	–4.36	0.0001

CKD				

Per capita disposable income	–0.0006	0.0004	–1.58	0.1170


*Note*: LKD: *R*^2^ = 0.1205, *R*adj^2^ = 0.1085; CKD: *R*^2^ = 0.0252, *R*adj^2^ = 0.0118.

## 4. Discussion

This study covered 85.4% (280/328) of KD-endemic counties in 15 KD-endemic provinces in China. Such wide range of surveillance was able to reflect the latest status of KD prevalence. Moreover, this study was conducted using a design of key investigation based on case-searching to ensure accuracy, using only results with the greater probability to find the endemic areas with the most severe prevalence. Furthermore, this was a nationwide county-level spatial epidemiological study of Keshan disease in China, which is small area study has the advantages of more reliable and geographically precise.

As shown in Figure 3A, CKD prevalence was randomly distributed and was not globally clustered, in contrast to the results of previous studies showing spatial clustering of CKD prevalence in China [32]. This difference indicates that KD prevention and control in China have significantly improved. At the present stage, most of the KD-endemic counties meet the standard of KD elimination, and only sporadic cases of CKD exist in several areas. This could explain why the global autocorrelation analysis showed that CKD prevalence was not globally clustered. Meanwhile, LKD prevalence had a positive spatial autocorrelation and was globally clustered, as shown in Figure 3B. These results might be explained by the following reasons. First, previous studies have shown that KD incidence is highly associated with selenium deficiency [33,34,35], and selenium levels in food and the environment are likely to be similar between neighboring counties. Second, it was found that income levels are associated with KD prevalence [13], and neighboring counties may share similar socio-economic backgrounds. Therefore, despite great achievements in KD prevention and control, LKD still exists and endangers the health of people living in some endemic areas, and consequently, KD prevention and control still cannot be ignored.

Global autocorrelation analysis can only be used to determine spatial clustering at the overall level and therefore cannot identify the specific areas and types of spatial clustering. Thus, local autocorrelation analysis must be conducted. The hot spots of LKD prevalence with statistical significance (95% CI and 99% CI) using local Getis-Ord Gi^*^ analysis were mainly observed in most counties of Shaanxi Province and Gansu Province in northwestern China, and in a few counties in Shanxi Province, Inner Mongolia Autonomous Region, and Jilin Province in northern China, as shown in Table 2 and Figure 5. These counties should therefore be the target areas for KD prevention and control. In the local Moran’s *I* analysis shown in Table 1 and Figure 4, H-H clustered areas indicated that the high values of LKD prevalence were clustered among neighboring counties (positive correlation). We found that H-H cluster areas were effectively detected in most counties of Shaanxi Province in northwestern China and in a few counties in Shanxi Province and Inner Mongolia Autonomous Region in northern China, Jilin Province in northeastern China, and Gansu Province in northwestern China. Thus, these areas should be prioritized to achieve KD precise prevention and control. Meanwhile, L-L cluster areas indicated that the low values of LKD prevalence were clustered among neighboring counties (positive correlation). Our findings showed that L-L cluster areas were detected in the counties of Sichuan and Chongqing in southwestern China. According to the results of our local autocorrelation analysis, KD prevention and control could be tailored to achieve spatially, geographically precise and visualized strategies for key endemic counties.

Furthermore, our spatial interpolation analysis helped in creating a map estimating the LKD prevalence in the endemic counties with missing data, as shown in Figure 6. The spatial interpolation analysis showed high LKD prevalence in most counties of Gansu, Shaanxi, and Shanxi Provinces and in a few counties of Jilin and Yunnan Provinces. The prevalence was lower in other counties, most of which were close to zero. These findings could provide spatially visualized evidence for formulating strategies for precision prevention and control of Keshan disease at the national level.

As shown in Table 3, LKD prevalence was significantly negatively correlated with per capita disposable income, while there was no significant correlation between CKD prevalence and per capita disposable income. This was consistent with the results of local spatial analysis of LKD prevalence, that the hot spots or H-H clustered areas were found in some endemic areas with poor economic conditions. Selenium deficiency has been well recognized to play a major role in the etiology of Keshan disease [5]. The selenium nutritional levels in KD endemic counties were statistically significantly lower than KD non-endemic counties [3]. Previous studies have reported that the income of residents was a considerable factor affecting nutritional intake. The intake of the major nutrients tended to increase with higher income, and families with different incomes had evident differences in the composition of nutrients in their diets [36,37]. These suggest that economic development is extremely important for KD prevention and control. The prevalence of LKD and CKD decreased by 0.0099 and 0.0006, respectively, for each unit increase in per capita disposable income. These results supported the etiological evidence from the etiological perspective of the remote cause of the causal chain of Keshan disease. The reason may be that increasing per capita disposable income can improve the diet structure and intake of nutrients, thereby meet the body’s nutritional needs and effectively enhance the body’s ability to resist disease.

The major innovations of this study are firstly the translation of the techniques of spatial statistical analysis into the practice of Keshan disease prevention and control. Secondly, this was a large-scale nationwide study using a design of key investigation based on case-searching at the county level, and we explored whether Keshan disease was clustered at the county level in order to provide the geographically visualized and precise evidence for the strategy of KD precision prevention and control. The limitation of this study was that the spatial regression model with LKD prevalence only included indirect indicators such as per capita disposable income. In terms of etiology, our further research is to measure the selenium biomarkers such as serum selenoprotein P, serum selenium, and hair selenium, which are the most representative of the body’s selenium nutritional level.

## 5. Conclusions

LKD prevalence was negatively correlated with per capita disposable income. Spatial analysis at the county level effectively identified the cluster areas of LKD prevalence in the provinces of Shaanxi, Gansu, Shanxi, Inner Mongolia, and Jilin. The H-H cluster areas should be given high priority for KD precision prevention and control.

## Abbreviations

KD, Keshan disease; CKD, chronic Keshan disease; LKD, latent Keshan disease; CI, confidential interval; GDP, gross domestic product; H-H, high-high; H-L, high-low; L-H, low-high; L-L, low-low.
